# Highlights of the Brazilian Society of Dermatology’s Brazilian Consensus on Psoriasis^؆^

**DOI:** 10.1016/j.abd.2025.501242

**Published:** 2025-11-10

**Authors:** Ricardo Romiti, André V.E. de Carvalho, Juliana Nakano, Gleison V. Duarte, Grupo de Trabalho do Consenso Brasileiro de Psoríase da Sociedade Brasileira de Dermatologia

**Affiliations:** a Department of Dermatology, Hospital das Clínicas, Universidade de São Paulo, São Paulo, SP, Brazil; b Hospital Moinhos de Vento, Porto Alegre, RS, Brazil; c Dermatology Clinic, Santa Casa de Misericórdia, São Paulo, SP, Brazil; d Instituto Bahiano de Imunoterapia, Salvador, BA, Brazil

**Keywords:** Algorithms, Consensus, Psoriasis

## Abstract

The 2024 Brazilian Consensus on Psoriasis and Treatment Algorithm of the Brazilian Society of Dermatology (SBD, *Sociedade Brasileira de Dermatologia*) comes four years after the release of the last Consensus, published in 2020. This new consensus now includes 43 chapters and has the participation of 85 professionals from across Brazil, including dermatologists and rheumatologists working in university hospitals, research centers, and public and private healthcare systems. The concept of Cumulative Life Course Impairment (CLCI), the inclusion of new biologics approved in Brazil after 2020, the update on psoriasis treatment in specific situations (such as pregnancy and surgery), and the discussion on "Response Predictors" are examples of updates addressed in this Consensus. The updated treatment algorithm used experts’ responses through the Delphi method and, among other modifications, removed acitretin from the plaque psoriasis treatment sequence, defined treatment lines for choosing immunobiologicals in both adult and pediatric populations, and included spesolimab and cyclosporine as first-line treatments for generalized pustular psoriasis. Finally, the new Consensus addresses the place of small molecule inhibitors in the algorithm and discusses future treatment options for plaque psoriasis.

The Brazilian Consensus on Psoriasis 2024 and Treatment Algorithm of the Brazilian Society of Dermatology (SBD) comes four years after the release of the last "Brazilian Consensus on Psoriasis 2020,"^1^ a period during which significant advances and innovations in the study of psoriasis were made, making updating the topic essential.

To develop this new work,^2^ which now includes 43 chapters, the authors relied on the participation of 85 experts from across Brazil, including dermatologists and rheumatologists from a wide range of regions (Fig. 1) and working in university hospitals, research centers, and public and private healthcare systems.

**Fig. 1:**
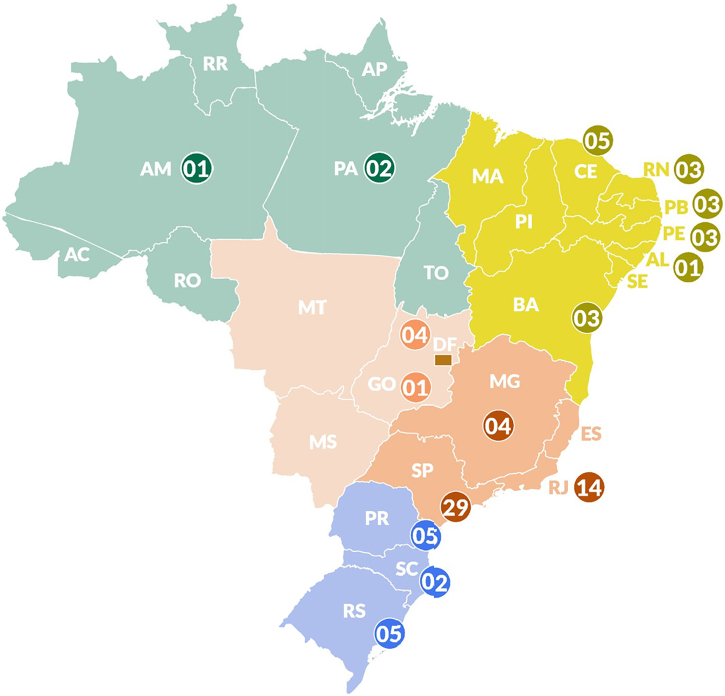
Distribution of the 85 specialists participating in the Brazilian Psoriasis Consensus 2024 according to the different states of Brazil.

Among the updates to the 2024 Consensus, the authors highlight the introduction of the concept of CLCI (Cumulative Life Course Impairment), which characterizes the set of factors detrimental to the lives of patients with psoriasis resulting from stigma and physical and psychological disability, as well as different coping strategies.^3^ Furthermore, the inclusion of new biological drugs and small molecules, such as bimekizumab, brodalumab, deucravacitinib, spesolimab, tildrakizumab, and deucravacitinib. The new Consensus also highlights specific situations such as neoplasms, surgeries, and other procedures. It also innovates with the discussion of "Response Predictors" to contribute to and personalize psoriasis treatment and optimize results for each patient.

This Consensus maintained the Delphi methodology adopted in the 2020 consensus,^1^ which facilitates decision-making by seeking to standardize approaches to controversial issues in the literature.^4^, ^5^ For this purpose, two rounds of questions were conducted, with anonymous participation from 80 and 76 authors, respectively, in each round. To ensure data validity, important criteria were adhered to, such as an adequate number of participants, representation from different regions of the country, the inclusion of experts from both the public and private healthcare systems, and the consistency of opposing questions in the same round. These measures help reduce potential biases. The consensus was considered reached when, for each question, at least 70% of voters assigned a score higher than 7 (Fig. 2).

**Fig. 2:**
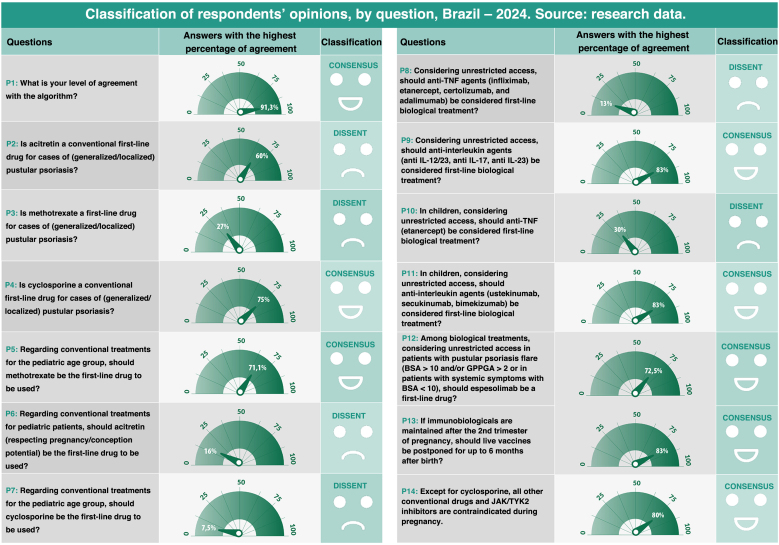
Classification of respondents’ opinions, by question, Brazil – 2024. Source: research data.

Finally, this Consensus updates the psoriasis treatment algorithm. For this purpose, the experts' responses were taken into consideration, and through the Delphi method, they reached an agreement on significant modifications compared to the 2020 Consensus. Among the new features of the updated severe psoriasis treatment algorithm, the authors highlight the removal of acitretin from the plaque psoriasis treatment sequence, with its use being reserved only for special situations. Deucravacinitib was also included in the treatment sequence, and treatment lines were defined for the selection of immunobiologicals. Anti-interleukin biologics were placed in the first-line position in relation to anti-TNF drugs. The treatment of psoriasis in the pediatric population was also included in the algorithm, with first- and second-line biologic treatments being defined. The algorithm (Fig. 3) includes a treatment sequence for flare-ups of generalized pustular psoriasis, with spesolimab and cyclosporine in the first-line therapy in this situation.

**Fig. 3:**
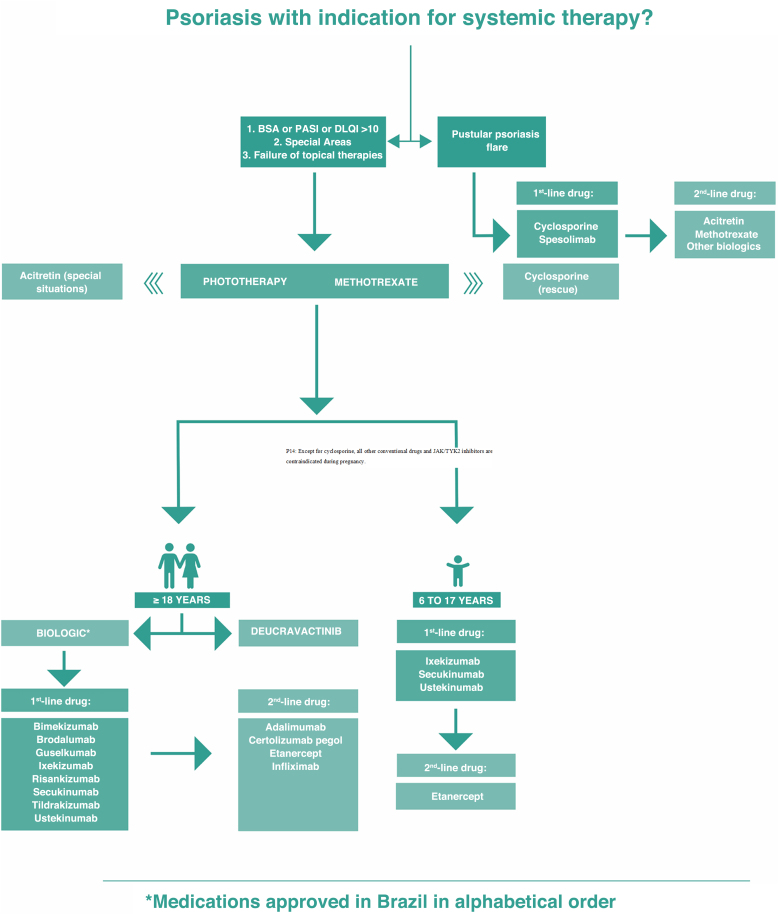
Treatment algorithm for severe psoriasis of the Brazilian Society of Dermatology.

In addition to the recently approved targeted therapies described in the new Brazilian Psoriasis Consensus, new pathophysiological pathways have been targeted for blocking with biologics and small molecules, offering new future therapeutic perspectives.

Recently, pivotal studies involving the use of an oral peptide antagonist of interleukin receptors involved in the inflammatory cascade of psoriasis promise to offer another innovative, potentially effective, and safe treatment line for the management of this immune-mediated dermatosis. The outlook for interventions in psoriasis seems promising, the safety profile of new treatments has proven satisfactory, and the debate about a possible cure for psoriasis is more current than ever.^6^, ^7^, ^8^ Future updates to this consensus are therefore mandatory, allowing the discussion of increasingly effective and safe measures in the management of patients with psoriasis.

## ORCID IDs

Ricardo Romiti: 0000-0003-0165-3831; André V.E. de Carvalho: 0000-0002-0407-538X; Juliana Nakano: 0000-0002-5638-3974

## Authors' contributions

Ricardo Romiti: Data curation; original draft; review and editing.

André V.E. de Carvalho: Data curation; original draft; review and editing.

Juliana Nakano: Data curation; original draft; review and editing.

Gleison V. Duarte: Data curation; original draft; review and editing.

Brazilian Society of Dermatology's Brazilian Psoriasis Consensus Working Group: Data curation; original draft.

## Funding

Brazilia Society of Dermatology.

## Research data availability

The entire dataset supporting the results of this study was published in this article.

## Conflicts of interest

Ricardo Romiti: Consultant, research, and speaker activities for AbbVie, BMS, Boehringer-Ingelheim, Galderma, J&J, LeoPharma, Lilly, Novartis, Pfizer, Sanofi, SunPharma, and UCB.

André V. E. de Carvalho: Participated as a speaker, consultant, participant in medical events, or researcher with the following companies: AbbVie, BMS, Boehringer-Ingleheim, GSK, Johnson & Johnson, Leo Pharma, Lilly, Novartis, Sun Pharma, and UCB.

Juliana Nakano: Participated as a speaker, consultant, participant in medical events, or researcher with the following companies: AbbVie, BMS, Eli Lilly, Johnson & Johnson, Sanofi, and UCB.

Gleison V. Duarte: Participated as a speaker, consultant, participant in medical events or researcher with the following companies: Abbvie, BMS, GSK, Amgen, Boehringer-Ingleheim, Galderma, Johnson&Johnson, Leo Pharma, Lilly, Novartis, Sun Pharma, Celldex Therapeutics and UCB.

## Appendix A Co-authors: Brazilian Society of Dermatology's Brazilian Consensus on Psoriasis Working Group

Adriana M. Porro

ORCID ID: 0000-0003-0736-4790

*Affiliation:* Escola Paulista de Medicina, Universidade Federal de São Paulo, São Paulo, SP, Brazil.

Adriane Reichert Faria

ORCID ID: 0000-0002-1020-7211

*Affiliation:* Irmandade Santa Casa de Misericórdia de Curitiba, Curitiba, PR, Brazil.

Pontifícia Universidade Católica do Paraná, Curitiba, PR, Brazil

Aldejane Gurgel de A. Rodrigues

ORCID ID: 0009-0008-6394-5868

*Affiliation:* Universidade de Pernambuco, Recife, PE, Brazil

Alexandre Gripp

ORCID ID: 0000-0003-2217-6704

*Affiliation:* Hospital Universitário Pedro Ernesto, Universidade do Estado do Rio de Janeiro, Rio de Janeiro, RJ, Brazil

Aline Bressan

ORCID ID: 0000-0002-3296-5232

*Affiliation:* Hospital Universitário Pedro Ernesto, Universidade do Estado do Rio de Janeiro, Rio de Janeiro, RJ, Brazil

Aline Okita

ORCID ID: 0000-0002-3796-137X

*Affiliation:* Universidade de Mogi das Cruzes, Mogi das Cuzes, SP, Brazil.

Ana Carolina Belini Bazán Arruda

ORCID ID: 0000-0002-0371-2308

*Affiliation:* Pontifícia Universidade Católica de Campinas, Campinas, SP, Brazil

Anber A. Tanaka

ORCID ID: 0000-0003-0963-0837

*Affiliation:* Faculdade Evangélica Mackenzie do Paraná, Curitiba, PR, Brazil

*Conflicts of interest:* Abbvie – conselho consultivo, consultor, palestrante, pesquisador; Boehringer-Ingelheim – conselho consultivo, consultor, palestrante, pesquisador; Eli-Lilly – conselho consultivo, consultor, palestrante, pesquisador; Janssen – conselho consultivo, consultor, palestrante; Leo Pharma – conselho consultivo, consultor, palestrante; Novartis – conselho consultivo, consultor, palestrante; pesquisador; UCB Biopharma – conselho consultivo, consultor, palestrante; Pfizer – palestrante, conselho consultivo; Sanofi – palestrante.

André Luís da Silva Hirayama

ORCID ID: 0000-0002-6276-3551

*Affiliation:* Faculty of Medicine, Hospital das Clínicas, Universidade de São Paulo, São Paulo, SP, Brazil

André V.E. de Carvalho

ORCID ID: 0000-0002-0407-538X

*Affiliation:* Hospital Moinhos de Vento, Porto Alegre, RS, Brazil

Andréa Machado C. Ramos

ORCID ID: 0000-0001-7414-3395

*Affiliation:* Service of Dermatology, Hospital das Clínicas, Universidade Federal de Minas Gerais, Belo Horizonte, MG, Brazil

Andréia Castanheiro da Costa

ORCID ID: 0000-0003-1732-3191

*Affiliation:* Centro Universitário Faculdade de Medicina do ABC, Santo André, SP, Brazil

Aripuanã Terena

ORCID ID: 0000-0002-2550-1341

*Affiliation:* Centro de Referência de Fototerapia e Fotobiologia Cutanea, Belo Horizonte, MG, Brazil

Arnóbio da Penha Pachêco

ORCID ID: 0000-0002-6904-7929

*Affiliation:* Hospital Universitário Onfre Lopes, Universidade Federal do Rio Grande do Norte, Natal, RN, Brazil.

Bethânia Cabral Cavalli Swiczar

ORCID ID: 0000-0003-2201-5628

*Affiliation:* IAMSPE and Complexo Hospitalar Padre Bento de Guarulhos, Guarulhos, SP, Brazil

Bruna Elena Graciano Falcone

ORCID ID: 0000-0002-0467-6791

*Affiliation:* ABC Faculty of Medicine, São Paulo, SP, Brazil

Cacilda da Silva Souza

ORCID ID: 0000-0002-8157-7658

*Affiliation:* Department of Internal Medicine, Division of Dermatology, Ribeirão Preto Faculty of Medicine, Universidade de São Paulo, Ribeirão Preto, SP, Brazil

Caio Cesar S. de Castro^a,b^

ORCID ID: 0000-0001-9767-5765

*Affiliation:* ^a^ Hospital de Dermatologia Sanitária do Paraná, Piraquara, PR, Brazil

^b^ Pontifícia Universidade Católica do Paraná, Curitiba, PR, Brazil

Carlos Eduardo de Mathias Sanches

ORCID ID: 0000-0003-3459-2414

*Affiliation:* São José do Rio Preto Faculty of Medicine, São José do Rio Preto, SP, Brazil

Catarina Santos

ORCID ID: 0009-0005-0010-7777

*Affiliation:* Universidade Estadual de Ciências da Saúde de Alagoas, Maceió, AL, Brazil

Ciro Martins Gomes

ORCID ID: 0000-0002-3069-6884

*Affiliation:* Postgraduate Program in Medical Sciences, Faculty of Medicine, Universidade de Brasília, Brasília, DF, Brazil

Clarice Marie Kobata

ORCID ID: 0000-0001-9563-0707

*Affiliation:* Irmandade Santa Casa de Misericórdia de São Paulo, São Paulo, SP, Brazil

Cláudia Pires Amaral Maia

ORCID ID: 0000-0003-3264-1518

*Affiliation:* Policlinica Geral do Rio de Janeiro, Rio de Janeiro, RJ, Brazil

Claudio Lerer

ORCID ID: 0000-0002-0229-196X

*Affiliation:* Hospital Universitário de Brasília, Brasília, DF, Brazil

Clivia Maria Oliveira

ORCID ID: 0000-0003-0406-360X

*Affiliation:* Universidade Federal do Pará, Belém, PA, Brazil

Cynthia Cristina Ferreira Mota

ORCID ID: 0000-0002-3745-2559

*Affiliation:* Specialty Outpatient Clinic, Psoriasis Sector, Santos City Hall, Santos, SP, Brazil

Daniel Holthausen Nunes

ORCID ID: 0000-0002-1303-5419

*Affiliation:* Universidade Federal de Santa Catarina, Florianópolis, SC, Brazil

Dimitri Luz

ORCID ID: 0000-0003-0869-9330

*Affiliation:* Universidade Santo Amaro, São Paulo, SP, Brazil

Domingos Jordão Neto

ORCID ID: 0000-0001-6244-7981

*Affiliation:* Hospital Heliópolis, São Paulo, SP, Brazil.

Eduardo Lacaz Martins

ORCID ID: 0000-0003-3563-9827

*Affiliation:* ABC Faculty of Medicine, São Paulo, SP, Brazil

Esther Bastos Palitot

ORCID ID: 0000-0002-8195-2534

*Affiliation:* Centro de Pesquisa, Apoio e Tratamento de Psoriase da Paraíba, Universidade Federal da Paraíba, João Pessoa, PB, Brazil

Fabrício Lamy

ORCID ID: 0009-0004-0214-5737

*Affiliation:* Policlínica Geral do Rio de Janeiro, Rio de Janeiro, RJ, Brazil

Francisca Regina O. Carneiro

ORCID ID: 0000-0001-6735-4004

*Affiliation:* Universidade do Estado do Pará, Belém, PA, Brazil

Gladys A. Martins

ORCID ID: 0000-0001-9913-2238

*Affiliation:* DermaCampbell, Brasília, DF, Brazil

Gleison V. Duarte

ORCID ID: 0000-0003-4160-5447

*Affiliation:* Dermatology Clinic, Instituto Bahiano de Imunoterapia, Salvador, BA, Brazil

Heitor de Sá Gonçalves

ORCID ID: 0000-0001-9062-2580

*Affiliation:* Centro de Referência Nacional em Dermatologia Sanitária Dona Libânia, Health Secretariat, Governo do Estado do Ceará, Fortaleza, CE, Brazil.

Jane Neffá

ORCID ID: 0000-0002-7888-0154

*Affiliation:* Hospital Universitário Antônio Pedro, Universidade Federal Fluminense, Niterói, RJ, Brazil

Jaquelini Barboza da Silva

ORCID ID: 0000-0002-5877-4290

*Affiliation:* Universidade de Santa Cruz do Sul, Santa Cruz do Sul, RS, Brazil

João Carlos R. Avelleira^a,b^

ORCID ID: 0000-0003-4683-7909

*Affiliation:* ^a^ Hospital Federal da Lagoa, Rio de Janeiro, RJ, Brazil

^b^ Instituto de Dermatologia Prof.Azulay, Santa Casa de Misericórdia, Rio de Janeiro, RJ, Brazil

Juliana Catucci Boza^a,b^

ORCID ID: 0000-0002-0573-1617

*Affiliation:* ^a^ Hospital de Clínicas de Porto Alegre, Universidade Federal do Rio Grande do Sul, Porto Alegre, RS, Brazil

^b^ Hospital Moinhos de Vento, Porto Alegre, RS, Brazil

Juliana Nakano^a,b,c^

ORCID ID: 0000-0002-5638-3974

*Affiliation:* ^a^ Irmandade Santa Casa de Misericórdia de São Paulo, São Paulo, SP, Brazil

^b^ Hospital Israelita Albert Einstein, São Paulo, SP, Brazil

^c^ Hospital Vila Nova Star, São Paulo, SP, Brazil

Juliana Viana Pinheiro Teixeira

ORCID ID: 0000-0002-8873-7095

*Affiliation:* Centro de Referência Nacional em Dermatologia Sanitária Dona Libânia, Health Secretariat, Governo do Estado do Ceará, Fortaleza, CE, Brazil

Juliana Yumi Massuda Serrano

ORCID ID: 0009-0000-7748-8583

*Affiliation:* Universidade Estadual de Campinas, Campinas, SP, Brazil

Leandro Linhares Leite

ORCID ID: 0000-0001-6370-3115

*Affiliation:* Hospital de Clínicas de Porto Alegre, Universidade Federal do Rio Grande do Sul, Porto Alegre, RS, Brazil

Leticia Oba Galvão

ORCID ID: 0000-0003-4486-1892

*Affiliation:* Hospital Regional da Asa Norte, Brasília, DF, Brazil

Ligia Pessoa de Melo^a,b^

ORCID ID: 0009-0006-5446-6860

*Affiliation:* ^a^ Hospital Otávio de Freitas, Recife, PE, Brazil

^b^ Instituto de Medicina Integral Professor Fernando Figueira, Recife, PE, Brazil

Lincoln Fabricio

ORCID ID: 0000-0001-9191-1085

*Affiliation:* Faculdade Evangélica Mackenzie do Paraná, Curitiba, PR, Brazil

Livia Barbosa

ORCID ID: 0000-0003-0326-1225

*Affiliation:* Private Prcatice Barbosa Souto Dermatologia, Rio de Janeiro, RJ, Brazil

Lucas Campos Garcia

ORCID ID: 0000-0002-7883-5986

*Affiliation:* Universidade Federal de Minas Gerais, Belo Horizonte, MG, Brazil

Luciane Donida Bartoldi Miot

ORCID ID: 0000-0002-2388-7842

*Affiliation:* Faculty of Medicine, Universidade Estadual Paulista, Botucatu, SP, Brazil

Luciena C. Martins Ortigosa

ORCID ID: 0000-0002-3541-9456

*Affiliation:* Clínica Triune, Presidente Prudente, SP, Brazil

Luis Antonio de Paula Machado

ORCID ID: 0009-0007-0551-7286

*Affiliation:* Hospital BP Mirante, São Paulo, SP, Brazil

Luíza Keiko Matsuka Oyafuso

ORCID ID: 0000-0003-3982-3322

*Affiliation:* ABC Faculty of Medicine, São Paulo, SP, Brazil

Luna Azulay-Abulafia^a,b^

ORCID ID: 0000-0002-4698-2009

*Affiliation:* ^a^ Universidade do Estado Rio de Janeiro, Rio de Janeiro, RJ, Brazil

^b^ Instituto de Dermatologia Professor Rubem David Azulay, Irmandade Santa Casa de Misericórdia do Rio de Janeiro, Rio de Janeiro, RJ, Brazil

Marcelo Arnone

ORCID ID: 0000-0002-7192-5161

*Affiliation:* Department of Dermatology, Universidade de São Paulo, São Paulo, SP, Brazil

Marcelo Pinheiro

ORCID ID: 0000-0002-1896-8322

*Affiliation:* Escola Paulista de Medicina, Universidade Federal de São Paulo, São Paulo, SP, Brazil

Marco Túlio Cavalcante Oliveira

ORCID ID: 0000-0002-9038-4911

*Affiliation:* Service of Dermatology, Hospital Universitário Walter Cantídio, Universidade Federal do Ceará, Fortaleza, CE, Brazil

Maria Cecilia de C. Bortoletto

ORCID ID: 0000-0002-6156-6097

*Affiliation:* Psoriasis Outpatient Clinic, Hospital do Servidor Público Municipal, São Paulo, SP, Brazil.

Maria de Fátima S. Paim de Oliveira

ORCID ID: 0000-0002-8594-200X

*Affiliation:* Universidade Federal da Bahia, Salvador, BA, Brazil

Maria Denise F. Takahashi

ORCID ID: 0000-0001-8671-6680

*Affiliation:* Faculty of Medicine, Universidade de São Paulo, São Paulo, SP, Brazil

María Victoria Suárez

ORCID ID: 0000-0002-2614-6011

*Affiliation:* Hospital do Servidor Público Municipal de São Paulo, Municipal Health Secretariat, São Paulo, SP, Brazil

Mauricio Amboni Conti

ORCID ID: 0000-0002-3502-1384

*Affiliation:* Hospital e Maternidade Marieta Konder Bornhausen, Itajaí, SC, Brazil.

Mayra Ianhez

ORCID ID: 0000-0003-3604-3128

*Affiliation:* Hospital de Doenças Tropicais, Goiânia, GO, Brazil

Michelle dos Santos Diniz

ORCID ID: 0000-0002-9259-0807

*Affiliation:* Irmandade Santa Casa de Misericordia de Belo Horizonte, Belo Horizonte, MG, Brazil

Mônica Nunes de Souza Santos

ORCID ID: 0000-0003-0578-3270

*Affiliation:* Department of Dermatology, Faculty of Medicine, Universidade do Estado do Amazonas, Manaus, AM, Brazil

Paulo A. Oldani Felix

ORCID ID: 0000-0002-1055-0597

*Affiliation:* Hospital Federal dos Servidores do Estado, Rio de Janeiro, RJ, Brazil

Paulo Eduardo de Sá Gonçalves

ORCID ID: 0000-0001-7843-7497

*Affiliation:* Centro de Referência Nacional em Dermatologia Sanitária Dona Libânia, Health Secretariat, Governo do Estado do Ceará, Fortaleza, CE, Brazil

Ralph Vighi da Rosa

ORCID ID: 0000-0002-7608-3813

*Affiliation:* Department of Dermatology, Universidade Católica de Pelotas, Pelotas, RS, Brazil.

Raquel Bissacotti Steglich

ORCID ID: 0000-0001-6550-3715

*Affiliation:* Universidade de Joinville, Joinville, SC, Brazil.

Renata Ferreira Magalhães

ORCID ID: 0000-0001-9170-932X

*Affiliation:* Department of Internal Medicine, Universidade Estadual de Campinas, Campinas, SP, Brazil.

Renata Rodrigues

ORCID ID: 0000-0002-0246-1316

*Affiliation:* Hospital Universitário Lauro Wanderley, Universidade Federal da Paraíba, João Pessoa, PB, Brazil

Ricardo Romiti

ORCID ID: 0000-0003-0165-3831

*Affiliation:* Department of Dermatology, Hospital das Clínicas, Universidade de São Paulo, São Paulo, SP, Brazil.

Roberta Buense

ORCID ID: 0000-0003-4739-9699

*Affiliation:* Irmandade Santa Casa de Misericórdia de São Paulo, São Paulo, SP, Brazil.

Roberto Souto

ORCID ID: 0000-0001-7136-6008

*Affiliation:* Hospital Universitário Pedro Ernesto, Universidade do Estado do Rio de Janeiro, Rio de Janeiro, RJ, Brazil.

Rosana Lazzarini

ORCID ID: 0000-0002-4893-3593

*Affiliation:* Dermatology Clinic, Irmandade Santa Casa de Misericórdia de São Paulo, São Paulo, SP, Brazil

Samir de Figueiredo Azouz

ORCID ID: 0000-0002-8831-9926

*Affiliation:* Universidade Federal da Paraíba, João Pessoa, PB, Brazil.

Sidney Augusto da C. Costa

ORCID ID: 0000-0002-5187-431X

*Affiliation:* Psoriasis Outpatient Clinic of the City Hall of Natal, Natal, RN, Brazil

Silvio Alencar Marques

ORCID ID: 0000-0002-5512-4700

*Affiliation:* Department of Infectious Diseases, Dermatology, Diagnostic Imaging and Radiotherapy, Faculty of Medicine, Universidade Estadual Paulista, Botucatu, SP, Brazil

Sineida Berbert Ferreira

ORCID ID: 0000-0001-7261-1220

*Affiliation:* Centro Paranaense de Estudos em Dermatologia, Maringá, PR, Brazil

Sueli Carneiro^a,b^

ORCID ID: 0000-0001-7515-2365

*Affiliation:* ^a^ Universidade do Estado do Rio de Janeiro, Rio de Janeiro, RJ, Brazil

^b^ Universidade Federal do Rio de Janeiro, Rio de Janeiro, RJ, Brazil

Vanessa Lucilia Silveira de Medeiros

ORCID ID: 0000-0002-1445-587X

*Affiliation:* Universidade de Pernambuco, Recife, PE, Brazil.

Verônica Rodrigues Bogado Leite

ORCID ID: 0000-0002-0232-2420

*Affiliation:* Department of Dermatology, Hospital Universitário Antônio Pedro, Universidade Federal Fluminense, Niterói, RJ, Brazil

Vivianne Lira da C. Costa

ORCID ID: 0000-0002-2259-3236

*Affiliation:* Hospital Universitário Onofre Lopes, Universidade Federal do Rio Grande do Norte, Natal, RN, Brazil

Wagner Galvão

ORCID ID: 0000-0001-6846-992X

*Affiliation:* Clínica ProDerma São Paulo, SP, Brazil

Xinaida Taligare V. Lima

ORCID ID: 0000-0001-7999-6979

*Affiliation:* Universidade Federal do Ceará, Fortaleza, CE, Brazil
